# Reflectance, illumination, and appearance in color constancy

**DOI:** 10.3389/fpsyg.2014.00005

**Published:** 2014-01-24

**Authors:** John J. McCann, Carinna Parraman, Alessandro Rizzi

**Affiliations:** ^1^McCann ImagingArlington, MA, USA; ^2^Centre for Fine Print Research, University of the West of EnglandBristol, UK; ^3^Dipartimento di Informatica, Università degli Studi di MilanoMilano, Italy

**Keywords:** color constancy, measured appearance, sensations, high-dynamic-range (HDR) scenes, discounting illumination, 3-D test targets

## Abstract

We studied color constancy using a pair of identical 3-D Color Mondrian displays. We viewed one 3-D Mondrian in nearly uniform illumination, and the other in directional, nonuniform illumination. We used the three dimensional structures to modulate the light falling on the painted surfaces. The 3-D structures in the displays were a matching set of wooden blocks. Across Mondrian displays, each corresponding facet had the same paint on its surface. We used only 6 chromatic, and 5 achromatic paints applied to 104 block facets. The 3-D blocks add shadows and multiple reflections not found in flat Mondrians. Both 3-D Mondrians were viewed simultaneously, side-by-side. We used two techniques to measure correlation of appearance with surface reflectance. First, observers made magnitude estimates of changes in the appearances of identical reflectances. Second, an author painted a watercolor of the 3-D Mondrians. The watercolor's reflectances quantified the changes in appearances. While constancy generalizations about illumination and reflectance hold for flat Mondrians, they do not for 3-D Mondrians. A constant paint does not exhibit perfect color constancy, but rather shows significant shifts in lightness, hue and chroma in response to the structure in the nonuniform illumination. Color appearance depends on the spatial information in both the illumination and the reflectances of objects. The spatial information of the quanta catch from the array of retinal receptors generates sensations that have variable correlation with surface reflectance. Models of appearance in humans need to calculate the departures from perfect constancy measured here. This article provides a dataset of measurements of color appearances for computational models of sensation.

## Introduction

Colorimetry and traditional color photography have fixed responses to spectral light. For each local area, the quanta catch of the light sensors determines the response of the system. For colorimetry, the quanta catch determines the match; and for silver-halide photography the quanta catch determines the optical density of the image. Their color processing has no color constancy. Humans sense their visual environment in real complex scenes. Humans have color constancy, such that appearance is largely indifferent to illumination. Further, they are insensitive to the changes in scene radiances due to shadows. This indifference in complex scenes is the result of spatial image processing. For centuries the best scene capture and rendering has been by artists who simply painted appearance. By simultaneously rendering the entire scene's spatial content in paint on a flat plane they recorded the visual equivalent of the real scene's greater range of light in nonuniform illumination.

In the late 19th century, discussions of constancy began with the study of the appearances of objects in different illuminations. In 1872 Hering wrote: “The approximate constancy of the colors of seen objects, in spite of large quantitative or qualitative changes of the general illumination of the visual field, is one of the most noteworthy and most important facts in the field of physiological optics. Without this approximate constancy, a piece of chalk on a cloudy day would manifest the same color as a piece of coal does on a sunny day, and in the course of a single day it would have to assume all possible colors that lie between black and white.”(Hering, 1872/1905).

**Figure 1:**
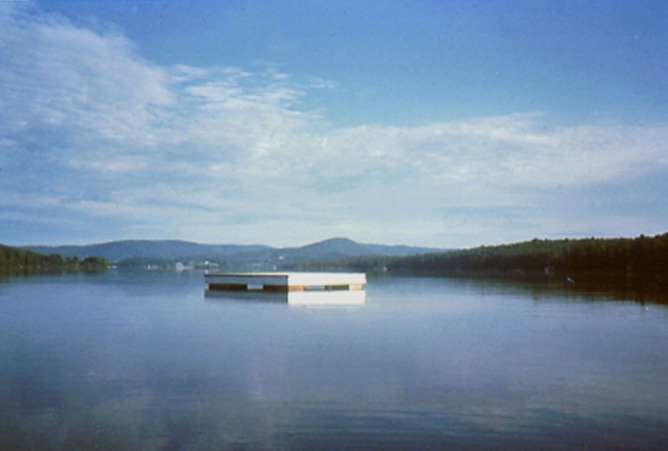
**Photograph of a swimming raft with sunlight illumination on the right and skylight illumination on the left**. Observers report sensations that are lighter and more yellow in the sun; and darker and more blue in skylight. Observers also report that the two sides of the float are perceived to have the same white paint, despite their different appearances.

At least four different kinds of color constancy are studied today. Although these disciplines have common roots, they have grown apart in their basic assumptions, terminology, and goals for successful implementation. These disciplines ask observers distinctly different questions and get answers that superficially seem to be contradictory. The Optical Society of America used a pair of definitions for sensation and perception that followed the ideas of the Scottish philosopher Thomas Reid. Sensation is a “Mode of mental functioning that is directly associated with the stimulation of the organism” (OSA Committee on Colorimetry, 1953). Perception is more complex, and involves past experience. Perception includes recognition of the object. It is helpful to compare and contrast these terms in a single image to establish our vocabulary as we progress from 18th century philosophy to 21st century image processing. Figure 1 is a photograph of a raft,—a swimming float—in the middle of a lake (McCann and Houston, 1983a; McCann, 2000a). The photograph was taken in early morning. The sunlight fell on one face of the raft, while the skylight illuminated the other face. The sunlit side reflected about 10 times more 3000°K light than the 20,000°K skylight side. The two faces had very different radiances, and hence very different colorimetric *X*, *Y*, *Z* values.

For sensation measurements, observers can select the colors they see from a lexicon of color samples, such as the Munsell Book, or the catalog of paint samples from a paint store. The question for observers is to find the paint sample that a fine-arts painter would use to make a realistic rendition of the scene. Observers say that a bright white with a touch of yellow looked like the sunlit side, and a light gray with a touch of blue looked like the sky-lit side. The answer to the sensation question was that the two faces were similar, but different.

For perceptions, observers can select the colors from the same catalog of paint samples, but with a different question. The perception question was to find the paint sample that a house painter would use to repaint the raft using the same paint. For this question, observers selected white paint. They recognized that the paint on both sides of the raft is white with different illuminations. The surface perception question renders the two faces identical.

In summary, the raft faces are very different, or similar, or identical depending on whether the experimenter is measuring colorimetry, or sensation, or perception. We need completely different kinds of image processing algorithms in order to model the three different answers to these three questions. Colorimetry models predict receptor responses; sensation models predict the color appearances; and perception models predict the observer's recognition of the object's surface. Subsequent experiments asked the same question, using a slightly different vocabulary (Arend and Goldstein, 1987). They found the same result. Namely, observer's responses depended on the observers' task.

### Color constancy models

Human color constancy involves the spatial content of the scene. As we will observe in this paper, it depends on the reflectances of objects, the spectral content and spatial distribution of the illumination, and the arrangement of the scene. There are a number of models of color constancy used to predict colors from the array of radiances coming to the eye, or the camera. They not only use a variety of image processing assumptions, they have different sets of required information, and different goals for the model to calculate. Table 1 lists the names of four types of models, their goals (result of the calculation), their required information (inputs to calculation), mechanisms, their dependence on surface reflectance and references (Table 1-row 1).

**Table 1:** **Four classes of color constancy models**.

**Model**	**Calculation goal [output]**	**Given information [model input]**	**Mechanism**	**Does output = Reflectance**	**References**
Retinex	Appearance (sensation)	Radiance array of entire scene	Build sensations from edges and gradients	Depends on scene content	Land and McCann, 1971
Discount illumination CIELAB, CIECAM	Appearance (sensation)	Pixel's radiance + pixel's irradiance	Measure reflectance stretch	Always	CIE, 1978 (CIELAB) CIE, 2004 (CIECAM)
Computer vision	Reflectance	Radiance array of entire scene	Estimate illumination to calculate surface	Always	Ebner, 2007; Gevers et al., 2012
Surface perception	Reflectance perception	Radiance array + adaptation	Cues, local adaptation, Bayesian inference	Depends on edges, adaptation and inference	Brainard and Maloney, 2004

#### Retinex

Land's Color Mondrian experiment (Land, 1964; Land and McCann, 1971) used a flat array of matte colored papers. He varied the amounts of uniform R, G, and B illumination over the entire array of more than 100 papers. He measured the light coming from a paper, then moved to a second paper and changed the illumination so that the second paper sent the same local stimulus to the eye. This experiment demonstrated that identical retinal stimuli can generate all colors. A red paper still looked red when its illumination was altered so that it was the same light stimulus as a green paper. The quanta catch of the retina at a pixel does not correlate with appearance. Color constancy measurements showed that color appearance correlates with the scaled integrated reflectance of the paper in Land's Color Mondrian (McCann et al., 1976). This good correlation uses Scaled Integrated Reflectance, not the usual spectral surface reflectance curves measured with a narrowband spectral radiometer. This integrated reflectance has L, M, S values that are the product of the spectral surface's reflectance, its irradiance, and the L, M, S retinal cone sensitivity functions. The L reflectance is the ratio of the L cone response to the surface divided by the Lcone response to an adjacent white paper in the same illumination. The scaling is done by the CIE L^*^ cube root function that approximates a correction for lower reflectances for scatter in the eye (McCann and Rizzi, 2012, ch. 14, 18).

McCann et al. (1976) calculated the paper's appearance using spatial comparisons. Further, cone sensitivity functions have considerable overlap. The L cones respond to middle-wave light, etc. The observed colors showed that the spatial comparison model predicted observer matches. The measured discrepancies from perfect constancy were predicted by crosstalk between the cone sensitivity spectra. (McCann et al., 1976; McCann, 2004c, 2005a).

Land's Retinex model requires, as input, the spectral radiances at each pixel in the field of view. Its goal is to calculate the appearance of all color sensations in the scene. It builds color appearances out of spatial comparisons. Land said “… the function of retinex theory is to tell how the eye can ascertain reflectance in a field in which the illumination is unknowable and the reflectance is unknown.” (Land and McCann, 1971). Later Retinex papers restated the language using edges and gradients, instead of illumination and reflectance. This was a result of studies of real life scenes in which: gradients in reflectance are difficult to see, and shadows with abrupt edges in illumination are highly visible (McCann, 1999b, 2004b).

Retinex, and other related models of vision, calculate sensations (McCann and Rizzi, 2012, p. 283–371). The correlation between surface reflectance and sensation depends on the scene's spatial content (Table 1-row 2).

#### CIELAB and CIECAM

Helmholtz proposed the idea that humans discount illumination, (von Helmholtz, 1866/1962) so that appearances correlated with recognizing the object, namely its reflectance. This principle is incorporated in pixel-based color appearance models such as 1976 CIELAB and 2002 CIECAM (CIE, 1978, 2004). These models use physical measurements of the illumination to normalize radiances from objects and remove the radiance information contained in the illumination. These models cannot predict color appearance without measurements of illumination at the pixel of interest as input. CIECAM requires that the user assign scene-dependent coefficients c (viewing condition parameter), N_c_ (chromatic surround induction factor), and F (surround parameter). These parameters have to be set by inspecting the scene (Moroney et al., 2002; Hunt, 2004). They are not calculated from the array of scene radiances (Table 1-row 3).

CIELAB and CIECAM use a pixel's scene radiance and that pixel's illumination. If two pixels from different parts of a scene have the same reflectance, but different illumination, then CIELAB and CIECAM predict identical outputs. These models predict that sensations always correlate with surface reflectances. CIELAB and CIECAM transform the color space of the scene radiances, but equal reflectances always generate equal sensations. There is no mechanism to introduce spatial variations caused by scene content.

#### Computer vision

Computer Vision Color Constancy algorithms work to remove the illumination measurement limitation found in CIE colorimetric standards by calculating illumination from scene data. The image processing community has adopted this approach to derive the illumination from the array of all radiances coming to the camera. Since estimating the illuminant from the pixel array is a multidimensional ill-posed problem, computer vision models need to apply some constraints on the scene. These constraints can regard spectral content or geometry of the illuminant, statistics of reflectances, etc. For example, one of the assumptions used in many Gray-World algorithms, is that scenes have a constant average reflectance (Buchsbaum, 1980; Funt and Drew, 1988). If true, then Gray-World algorithms can use the average radiance of all pixels to measure the spectral distribution of the illuminant.

As long as the illumination is constant for all pixels in the scene, then each pixel's radiance divided by the calculated illumination will equal that pixel's reflectance. Computer-vision models measure success by how well they can calculate an object's reflectance in different spectral illuminants. In order to use these models in a discussion about human vision, we need to perform a separate psychophysical experiment to test whether appearances correlate with reflectance for the image in question. One should not use such models for vision in situations where appearance deviates from reflectance. These models often assume perfect color constancy which is quite different from the approximate constancy found in humans. This field has been studied by Horn (1974), Buchsbaum (1980), Marr (1982), Funt and Drew (1988), Richards (1988), D'Zmura and Iverson (1993a,b), Sinha and Adelson (1993), Adelson and Pentland (1996), Finlayson et al. (1997), Purves and Lotto (2003), Zickler et al. (2008), Foster et al. (2009), Gevers et al. (2012) (Table 1-row 4).

Summary of Computer Vision Color Constancy are found in Ebner (2007) and Gevers et al. (2012). Many of these studies use shared datasets to optimize their algorithms. Instead of each experiment devoting the authors' resources to making complete sets of measurements of each phenomenon, computer vision research often collaborates by the use of shared data. Examples of datasets of images provided for other authors to test their algorithms are found in Grosse et al. (2009), and Gevers et al. (2012).

Color Constancy in Computer Vision searches for the object's intrinsic surface properties. That definition sets the algorithm's goal as finding surface reflectance. That goal implies the accurate calculation of illumination from the array of scene radiances.

#### Surface perception

Surface Perception algorithms study and model the observer's ability to recognize the surface of objects. Following Hering's concern that chalk should not be mistaken for coal, the objective is to predict human response to questions about recognizing an objects surface. Here the subjects are asked the house painter's question: what paint is on the surface? Techniques include the analysis of cues from specular reflections and application of Bayesian inferences. This field has been studied by Helson (1947, 1964), Lee (1986), Yang and Maloney (2001), Bloj et al. (2004), Brainard and Maloney (2004), Ripamonti et al. (2004), Smithson and Zaidi (2004), Brainard et al. (2006), Gilchrist (2006), Foster et al. (2009), Kingdom (2011).

Helson (1947, 1964) believed that the complex visual image generated a “pooled effect of all stimuli,” to which the organism was “attuned or adapted.” Helson's level of reference is centrally stored and used as reference for all judgments. Many elements of the visual environment are suggested to play a role in such a global normalization factor, such as visual pigment adaptation, the history of reflectances in the field of view, and temporal distribution of cues (Smithson and Zaidi, 2004). See Brainard and Maloney (2004) for summary (Table 1-row 4).

All four types of algorithms listed in Table 1 do well with their predictions in the flat uniformly illuminated Color Mondrian. As long as the illumination is uniform, the sensation predictions of Retinex and CIECAM models are similar. Further, sensations correlate with reflectance in uniform illumination (McCann et al., 1976). That has led some authors to suggest that Computer Vision algorithms can be used in modeling vision (Ebner, 2007). All four of the distinct Color Constancies listed in Table 1 involve the interpretation of scene radiances. Beyond that common variable, they differ in their use of reflectance, illumination, scene content, sensations, and perceptions.

The experiments in this paper introduce a different set of requirements for color appearance models. Here, we use a restricted set of reflectances and highly variable illumination. By varying the spatial structure in the illumination we have more realistic stimuli representing complex scenes, and we greatly increase the dynamic range of the scene. We have more information to help sort out the importance of radiance, reflectance and illumination, as well as scene content, including edges and gradients, in modeling human vision. By studying the effects of spatial structure in illumination we will attempt to compare and contrast the roles of reflectance and illumination in these four types of color constancy.

### Dataset of sensations—departures from perfect constancy

These experiments measure sensations. They ask the “fine arts painter” question. What is the appearance of the surface?

This article describes experiments that measure the departures from perfect constancy in complex scenes. If human color sensation constancy were perfect then the same surface paint would generate identical sensations in all illuminations and in all scenes. Human constancy is rarely perfect. It is observed only when the retinal quanta catches are constant in surrounding scenes that are identical. What is remarkable about human vision is how small the departures are in most scenes. We can measure these departures from perfect constancy to test computational models of sensations. In other words, departures from perfect constancy are the signature of the underlying mechanisms.

The much earlier McCann McKee and Taylor paper: (1) measured the sensations in flat 2-D Mondrians. They modeled observer results with: (2) measured scene radiances; (3) calculated cone responses; and (4) spatial algorithm calculations of color sensations. They successfully modeled the observer results. They found that appearances in *that* Mondrian correlated with the spectral measurements of reflectance using spatial comparisons (edge ratios) of cone responses.

We are in the process of performing the same steps here. However, our scenes are much more complicated. This paper intends to perform only the first step by collecting the dataset of departures. Other more complex steps will follow. This paper describes the measurement of a dataset of departures from perfect constancy in 3-D Mondrians. Here, the three dimensions of the target are used to modulate illumination. We are not studying the perceptual effects of depth perception. Here, the objects modify the illumination by introducing gradients and edges.

The process for calculating the cone quanta catch generated by these 3-D Mondrians is beyond the scope of this paper. Both cameras and human intraocular glare introduce different major spatial transformations of scene radiances measured by a telephotometer. You cannot use camera data, even RAW camera data, as an input to computational models of vision. Detailed calibrations are needed to prove that a particular digital camera image is an accurate record of the scene (McCann et al., 2013). As well, we need to use the CIE model of intraocular glare (Vos and van den Berg, 1999) to calculate the retinal image. Glare is a variable addition to scene radiances depending on scene content. Radiance measurements of the scene do not represent retinal stimuli, particularly in high-dynamic-range scenes (McCann and Rizzi, 2012, p. 113–171). As well, the implementation of spatial algorithms (McCann and Rizzi, 2012, p. 283–371) to calculate predictions of sensations from the retinal image is beyond the scope of this paper.

## Materials and methods

**Figure 2:**
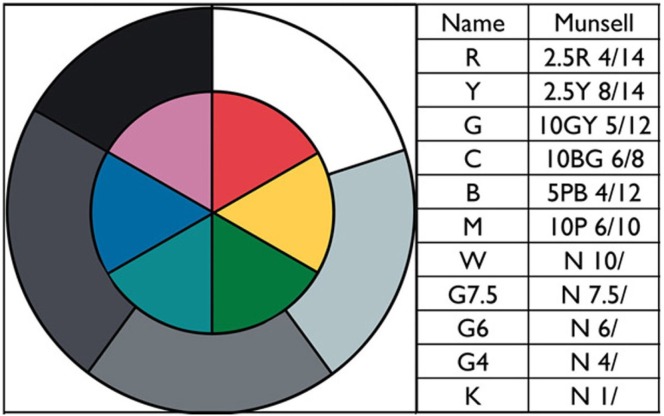
**“Ground truth” painted test target and the Munsell designations for the 11 paints**.

This article studies human color appearances of surface reflectances in real complex scenes. These scenes are made up of two copies of the same surfaces (wooded blocks with matte paints). There are only 11 paints (R, Y, G, C, B, M, W, G7.5, G6, G4, K). Figure 2 (left) shows a circular test target with 11 painted sections. Figure 2 (right) lists the Munsell chip closest to each paint, evaluated in daylight. The colors were selected among matte surface paints. The five grays were selected to maximum and minimum reflectances with two paints near middle gray, and a light gray. The six colors were selected to red—neither orange red, nor purple red; yellow—neither warm, nor cool yellow; … etc. They have high chroma, but not maximal chroma.

We worked with these 11 painted surfaces to construct two parts of a 3-D complex scene.

1. Low Dynamic Range (LDR) with as uniform illumination as possible from multiple tungsten lights (diffused in an illumination cube).
2. High Dynamic Range (HDR) with two directional lights with different spectra (White LED and Tungsten).

Both 3-D Mondrians were made of two sets of identical wooden blocks. They used the same paint on each corresponding facet. Both the LDR and the HDR parts of the scene were viewed in the same room at the same time. Ideally the LDR illumination would be perfectly uniform. That would restrict the range of scene radiances to the range of surface reflectances. While this is possible with flat Mondrians, measurements of surfaces in our LDR illumination cube showed a small range of nonuniformity.

HDR scenes are generated by directional light and the presence of light emitters. We use the terms LDR and HDR as labels of our experimental illumination, and they should not be confused with tone-mapping algorithms in digital photography.

By varying the illumination on constant surfaces we can measure the extent of color constancy of sensations. Does appearance correlate with the objects physical reflectance, or scaled integrated reflectance? How does appearance change with different illuminations? Does the spatial content of the illumination play a role in appearance?

All color appearance measurements were made on the combined LDR/HDR display described in Section LDR/HDR Display The first measurement set (Section Magnitude Estimation Color Appearance in the Munsell Book) were made using observer magnitude estimates of the changes in appearance with reference to distances in the Munsell Book. In the second measurement set (Section Artist's Rendering of Scene Appearances), an artist recorded the color appearances of all the blocks in a watercolor painting. We then measured the visible reflectance spectrum of each area's color match. By painting the entire scene we measured the appearance of a facet in the surround equivalent to that in the scene.

### LDR/HDR display

**Figure 3:**
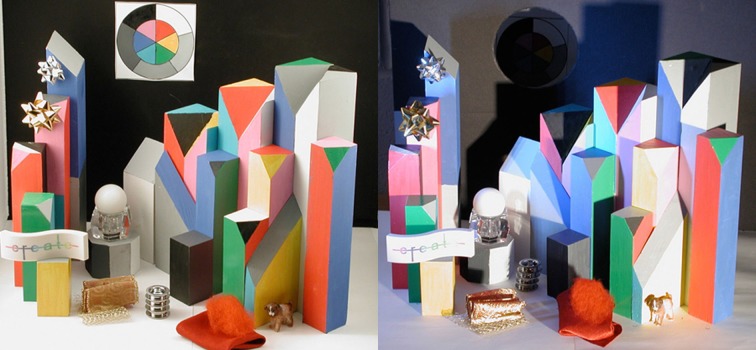
**Photographs of the Low-Dynamic-Range (LDR) part of the scene on left, and High-Dynamic-Range (HDR) on right**.

We made a pair of photographs of the two parts of the scene. Figure 3 (left) shows the LDR part. The blocks were inside an illumination cube with a white floor, translucent top and sides, and a black background. We directed eight tungsten-halide spotlights on the sides and top of the illumination cube. The combination of multiple lamps with identical emission spectra, light-scattering cloth of the illumination cube, and highly reflective walls made the illumination nearly uniform. Departures from perfect uniformity came from shadows cast by the 3-D objects, and the open front of the cube for viewing.

Figure 3 (right) is a photograph of the HDR 3-D Color Mondrian illuminated by two different lights. One was a 150 W tungsten spotlight on the right side of the HDR Mondrian at the same elevation. It was placed 2 m from the center of the target. The second light was an array of WLEDs assembled in a flashlight. It stood vertically and was located 20 cm from the display on the left. Although both illuminants are “white lights,” they have different emission spectra. The placement of these lamps produced highly nonuniform illumination and increased the dynamic range of the scene (McCann et al., 2009a,b). The overhead lights in the room were shut off, but the display provided sufficient working illumination.

In the HDR 3-D Mondrian, the black back wall had a 10 cm circular hole cut in it. Behind the hole was a small chamber with a second black wall 10 cm behind the first. We placed the flat circular test target on the back of this chamber. It was placed so that none of the direct light from either lamp fell on the circular target. That target was illuminated by light reflected by the black walls of the chamber. The target in the chamber had much less illumination than the same paints on the wooden blocks. The target in the chamber significantly increased the range of the nonuniform display. Nevertheless, observers had no difficulty seeing the darker circular target.

**Figure 4:**
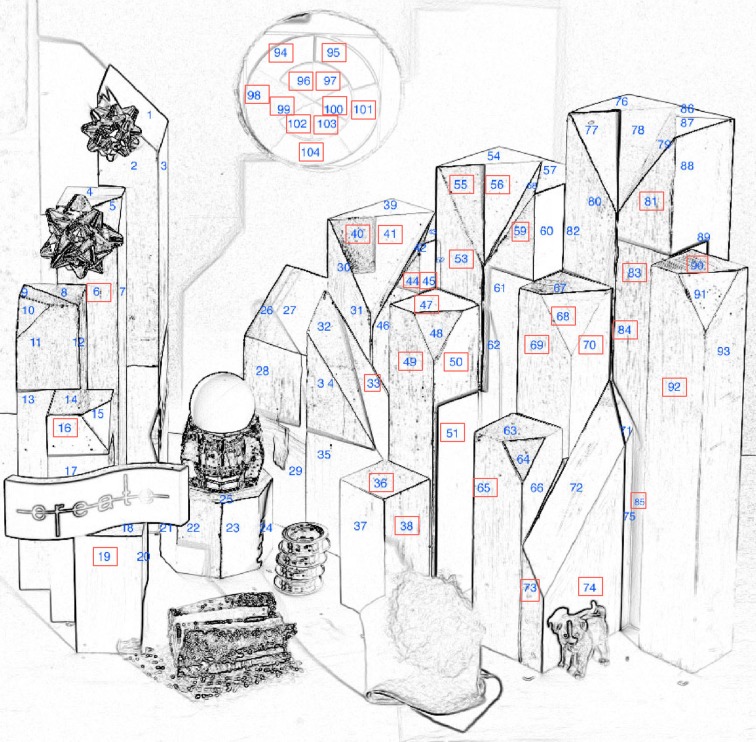
**Identification numbers for the 104 wooden block facets. The red rectangles identify the 37 facets evaluated by magnitude estimation**.

Figure 4 identifies the 104 painted facets measured in these experiments. This information is needed to identify the individual areas in the LDR and HDR displays. The highest luminance was 273 cd/m^2^. It was the white paint facet #60 in the HDR portion. The lowest HDR facet was 0.73 cd/m^2^ (black paint, facet #94), giving a range of 377:1.

In the LDR portion the highest luminance was 248 cd/m^2^ (white paint facet #9) and the lowest was 3.4 cd/m^2^ (black facet #21), giving a range of 74:1.

The range of luminances for white and black paint in uniform illumination is 17:1. We measured the radiances of each facet in both LDR and HDR parts of the target [Appendix 2 (Data Sheet)—data normalized and scaled].

### Magnitude estimation color appearance in the Munsell book

We asked 10 observers to measure the color appearances of identical painted surfaces. The average age of observers was 32; there were 6 males and 4 females. All observers reported that they had their color vision tested. They all had normal color vision. The experimental design was reviewed by the Ethical Committee of Università degli Studi di Milano.

Before the start of the experiment, we informed observers that they were comparing the appearance of wooden facets with identical painted surfaces. Each observer was given two documents. One was a data sheet to be used to record their responses. For example, the HDR part of the sheet had a color photograph of the HDR scene with five arrows pointing to five facets with R paint, labeled R1–R5. Adjacent to each arrow, there was a location for the observer to report the Hue, Lightness, and Chroma changes in appearance from “Ground Truth” of that red paint facet. The four-page data sheet had 9 LDR photographs, each with specific arrows identifying the facets to be evaluated for that paint. It also had 9 HDR photographs with arrows identifying the same facets. The four-page form identified a selection of 37 areas in both the LDR and HDR parts. The 37 LDR and HDR facets were chosen to represent examples of nine paints. Some were chosen to document the changes in appearance in the LDR part, and others for changes in appearance in the HDR part. Nine areas in the box behind the HDR circular hole were included.

**Figure 5:**
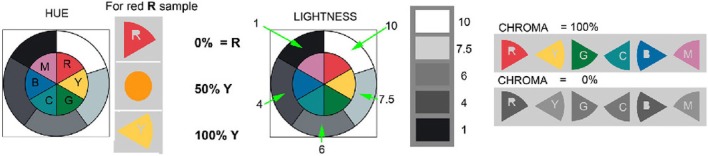
**The ground truth reflectance examples**. These diagrams were given to observers to describe the strategy for magnitude estimation of hue **(left)**; lightness **(center)**; chroma **(right)**.

The second paper handed each observer was a copy of Figure 5. It provided written guidance on their magnitude estimates. The observers were shown a pair of painted circular test targets (Figure 2 left) placed on the floor of each display, in uniform light. This circular array of the paints was defined to be the appearance of ground truth. They were told that all the flat surfaces had the same paints as those on the “ground truth” targets. We explained that we were asking about what the area looked like—its appearance—its sensation (OSA Committee on Colorimetry, 1953).

If the observer reported that a facet appeared the same as the red paint applied to the “ground truth” test target, we called that an example of perfect color constancy. In other words, the human did a perfect job of ignoring the illuminant. When a facet appeared different from the paint in the “ground truth” target, we asked the observer to estimate the change in sensation using guidelines reproduced in Figure 5.

Observers were asked: “Do the selected facets have the same appearance as ground truth?” If not, they described the direction and magnitude of the change in appearance using the following procedure. Observers estimated hue changes starting from each of the six patches of colors labeled R, Y, G, C, B, and M. The written instructions stated: “If the facet changes hue, estimate how much it moved toward another color?” They considered changes in the hue as a percentage between one hue (e.g., R), and another hue (e.g., Y). For example, 50%Y indicates a hue shift to a color halfway between R [Munsell 2.5R] and Y [Munsell 2.5Y] (Figure 5 left). 50%Y equals Munsell 2.5YR. 100%Y meant a total shift of hue to Y.

Observers estimated lightness differences on a Munsell-like scale indicating either increments or decrements, for the apparent lightness value (Figure 5, center). They were given the Munsell Lightness Values of *W* = 10; G7.5 = 7.5; G6 = 6; G4 = 4; *K* = 1. The instructions said: “If the facet looks the same lightness as the standard area G6, write down G6. If the facet looks lighter, or darker, estimate how much using the ground truth lightness values.” They were asked to estimate the apparent lightness of each area.

Observers estimated chroma by assigning paint sample data relative to ground truth defined as 100% (Figure 5 right). If the sample had the same chroma as ground truth then they gave the value 100%. Zero % was assigned to achromatic appearances. In case the target patch appeared more saturated than ground truth, estimates could be greater than 100% (Parraman et al., 2009).

To relate ground truth to the Munsell Book Notation, we matched the 11 painted ground truth samples, by placing Munsell chips on top of the paint samples in daylight. The Munsell notations of the 11 paints are listed in Figure 2.

Observers reported the direction and magnitude of changes in appearance from ground truth. We used linear scaling to calculate the Munsell designation of the matching Munsell chip. We used the distance in the Munsell Book as described in the MLAB color space, (Marcu, 1998; McCann, 1999a) as the measure of change in appearance. We assumed that the Munsell Book of Color is equally spaced in color. MLAB converts the Munsell designations to a format similar to CIELAB, but avoids its large departures from uniform color spacing (McCann, 1999a). When the observer reports no change in appearance from illumination MLAB distance is zero. A change as large as white to black (Munsell 10/ to Munsell 1) is MLAB distance of 90.

The results, presented in Appendix (Data Sheet) 1, are the average ± standard error of the mean of 10 observers' estimates of the selected areas in the pair of LDR and HDR 3-D Mondrians. We converted the observer magnitude estimates to an observed Munsell chip designation, and then to MLAB Color space. Munsell chips vary from less than 10 to 1, and L * a * b * varies from 100 to 0. We multiplied estimated Munsell Lightness Values by 10.

(1) ML=10∗(Munsell Lightness Value)

(2) Mb=5∗(Munsell Chroma∗sin(Hue Angle∗PI/180))

(3) $$Ma={\left({\left(5*\text{Chroma}\right)}^{2}+Mb^{2}\right)}^{0.5}$$

We multiplied Munsell Chroma by 5 and by the sine of the hue angle to calculate *Mb*. Ma is the third side of the triangle in the Chroma plane for that Lightness (McCann, 1999b). We averaged the 10 observed *ML*, *Ma*, *Mb* values for each chip. This representation of the data allows us to calculate distance in the uniform Munsell Space.

In McCann et al. (1976) observers matched rectangles in flat displays in uniform illumination to the Munsell Book. There the departures from perfect constancy were small. We used matches to the Munsell Book for greater accuracy. The average standard deviation for a match was close to ± one Munsell chip for this technique. In preparing these 3-D Mondrians, we observed how large the departures were. Some of them were as large as 60% of the range between white and black. Matching to Munsell chips is much slower, and more difficult with nonuniform illumination. We chose to use magnitude estimation techniques (Stevens, 1975; Bodmann et al., 1979) because they are more efficient. Although magnitude estimation increases the variance of measurements, the mean data is reliable and repeatable. Observers estimated the linear change in appearance in the uniformly spaced Munsell Book. Wyszecki and Stiles (1982, Appendix) provide a MacAdam table of *Y*, *x*, *y* values for Munsell Designations that extend to the spectrum locus. Thus, magnitude estimates can extend beyond the limitations of Munsell's samples. We chose to use magnitude estimation so we could increase the number of observers.

### Artist's rendering of scene appearances

After observers finished the Magnitude Estimations of Munsell designations, we left the pair of 3-D Mondrians in place. One of the authors (Carinna Parraman) painted with watercolors on paper a rendition of both 3-D Mondrians (Parraman et al., 2010). The painting took a long time to make the reproduction as close as possible to the appearances in both displays. Painters are usually applying their particular “aesthetic rendering” that is a part of their personal style. In this case the painter worked to present on paper the most accurate reproduction of appearances possible. As with the magnitude estimation measurements, both LDR and HDR were viewed and painted together in the same room at the same time.

We made reflectance measurements of the watercolor with a Spectrolino® meter in the center of the areas identified in Figure 4. We measured the reflectance spectra of both LDR and HDR watercolor paintings at each of the 104 facets. The meter reads 36 spectral bands, 10 nm apart over the range of 380–730 nm. We calibrated the meter using a standard reflectance tile.

We considered how to represent these reflectance measurements taking into account human vision. Analysis of percent reflectance overemphasizes the high-reflectance readings, while analysis using log reflectance (optical density) overemphasizes the low-reflectance values. Experiments that measure equal changes in appearance show that the cube root of reflectance is a good approximation of equal visual weighting (Wyszecki and Stiles, 1982). This nonlinear cube root transformation of reflectance has been shown to correlate with intraocular scatter (Stiehl et al., 1983; McCann and Rizzi, 2008). Thus, the cube root of scene luminance converts it to an approximation of log retinal luminance (McCann and Rizzi, 2009). Studies by Indow (1980), D'Andrade and Romney (2003) used the L^*^ transform as the first step in their studies of how cones, opponent processes, and lateral geniculate cells generate the perceptually uniform Munsell Color Space. We used the L^*^ function Equation (4) to scale Spectrolino reflectance values for each waveband.

(4) $${\text{L}}_{\lambda }^{*}=116*{\left({\text{reflectance}}_{\lambda }\right)}^{1\text{​}/3}-16$$

Appendix (Data Sheet) 2 lists the scaled XYZ transformations of reflectances of 11 ground truth paint samples: the radiances LDR and HDR facets; and the reflectances of the LDR and HDR watercolor paints. The middle of Appendix (Data Sheet) 2 lists the normalized radiance measurements made with a Konica Minolta CS100 colorimetric telephotometer. We measured (*Y*, *x*, *y*) for each block facet. They were converted to *X*, *Y*, *Z*; averaged and normalized in LDR by the White paint Area 9 measurements (*X* = 284.7, *Y* = 247.5 cd/m^2^, *Z* = 62.8); in HDR by the White paint Area 60 measurements (*X* = 314.2, *Y* = 273 cd/m^2^, *Z* = 88.5). These normalized values were scaled by L * Equation (4).

The color space used in the watercolor measurements describes the painted matches in the framework of retinal responses. It is the color space used in Colorimetry to represent the first step in color vision. The color space used in the Magnitude Estimation measurements is the end of the color process, namely the uniform color space of Munsell. In a uniform color space the retinal responses have been transformed by opponent-color processes to significantly expand chroma, and to counteract the effects of cone crosstalk.

## Results

The goal of these experiments is to evaluate how complex, nonuniform illumination affects color constancy. In the 3-D Mondrians, the objects in the scene modulate the illumination. It is a departure from our previous experimental design using uniform illumination that varies only in its spectra (McCann et al., 1976). We used two different techniques with different observers and different skills. The magnitude estimate experiment used the average of 10 observers to assess the changes in Munsell Color space using distance from ground truth and the direction of the departure from constancy in color space (Section Magnitude Estimates Results). The artist rendering of appearance in the watercolor makes a different comparison in a different color space. It measured the reflectance spectra of a matching image (Section Artist's Watercolor Appearances Results).

### Magnitude estimates results

We measured the departure in sensation from constancy in Munsell Space by calculating the observers' average *ML*, *Ma*, *Mb* magnitude estimate for each color paint. We used two circular targets, one in front of each part of the display as the ground truth starting point. For example: R matches Munsell chip 2.5R 4/14. We converted this Munsell designation to MLAB values (*ML* = 40, *Ma* = 70, *Mb* = 7). The red paint in the circular target on the back wall [Area 97] (See Figure 2, left), had an average observer appearance estimate of *ML* = 44.5, *Ma* = 66.5, *Mb* = 5.8 for LDR, and *ML* = 27.1, *Ma* = 59.4, *Mb* = 4.3 for HDR. We calculated the distance between average of observed sensations and ground truth as the square root of the sum of the squares of average Δ *ML*, Δ *Ma*, and Δ *Mb* differences (Table 2).

**Table 2:** **For facet 97, the list of the *ML*, *Ma*, *Mb* values of ground truth (top row); LDR, and HDR average magnitude estimates; their differences and the distances between them in Munsell Space and the direction of these departures in *ML* vs. *Ma*, and *Mb* vs. Ma planes**.

	***ML***	***Ma***	***Mb***	**Distance**	**Angle (*Ma*/*ML*)**	**Angle (*Ma*/*Mb*)**
Ground truth (gt)	40	70	7			
LDR	44.5	66.5	5.8			
HDR	27.1	59.4	4.3			
Δ(LDR-HDR)	17.4	7.1	1.5	18.9	248°	192°
Δ(LDR-gt)	4.5	−3.1	−1.5	5.7	125°	206°
Δ(HDR-gt)	−12.9	−10.2	−3	16.7	232°	196°

The distance between LDR and HDR average magnitude estimates was 18.9 MLAB units, or the equivalent of 20% of the distance between white and black in the uniform Munsell Color Space. The LDR appearance of facet 97 was 5.7 units away from ground truth moving in the direction of 125° in the *ML* vs. *Ma* plane; and moving in the direction of 206° from ground truth in the *Mb* vs. *Ma* plane.

The HDR appearance of facet 97 was 16.7 units away from ground truth moving in the direction of 232° in the *ML* vs. *Ma* plane; and moving in the direction of 196° in the *Mb* vs. *Ma* plane. We asked the observers to evaluate 5 facets with red paint in HDR. These results are listed in the top section of Appendix (Data Sheet) 1 dataset.

**Figure 6:**
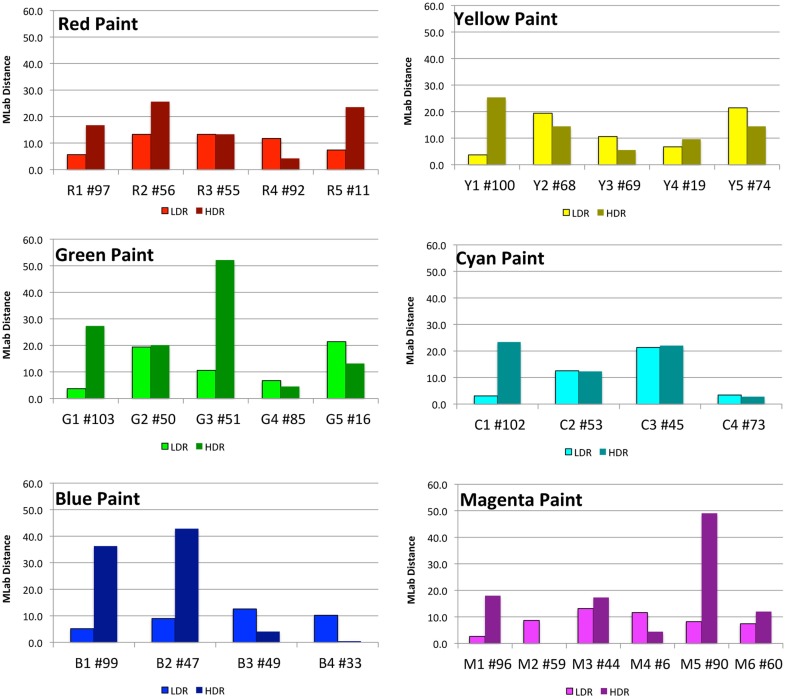
**Plots of distances in MLAB color space between observer match and ground truth, segmented by paint color**.

Appendix (Data Sheet) 1 lists the data described above for all color samples reported by observers. It includes multiple areas with the same painted surfaces. Figure 6 plots the MLAB distances from ground truth for the six chromatic paints. In general, these distances are greater in HDR illumination. However, for each color there is at least one sample that changes appearances more in the LDR than in the HDR illumination.

The lightness (*ML*), hue/chroma plane (*Ma*, *Mb*) for nine paints and the average magnitude estimates for 37 selected areas in both LDR and HDR are listed in Appendix (Data Sheet) 1. For each area, we list the average *ML*, *Ma*, *Mb* values; the change in appearance from ground truth as delta *ML*, delta *Ma*, delta *Mb*, and the MLAB distance in the Munsell Book. Appendix (Data Sheet) 1 also lists the ranges of for each paint sample and the angles of departures from constancy. The following detailed results will show observers reported larger departures from ground truth in the HDR than in the LDR scenes. We analyze the result from each set of nine paints. For the red paint the LDR ranges were Δ *ML* = 9, Δ *Ma* = 4, Δ *Mb* = 3; the HDR ranges were Δ *ML* = 25, Δ *Ma* = 26, Δ *Mb* = 24. This pattern held for all the paints. For the five red samples, the individual distances in Munsell MLAB space were LDR = 6, 13, 13, 12, 7 and HDR = 17, 25, 13, 4, 29. This illustrates an important point. In the LDR scene the changes in appearance were smaller in nearly uniform illumination. In the HDR scene the changes in appearance were larger, but there were individual areas that showed little or no change from ground truth. The changes in appearance in the LDR were larger in lightness than in hue/chroma. The changes in HDR were found in both lightness and hue/chroma. Area 11 in LDR is *ML* = 43, *Ma* = 64, *Mb* = 3. This is 3 units lighter, 5 units less red, and 4 units bluer than ground truth. In HDR illumination Area 11 is a sample of red paint that is close to the LED illumination on the right. It has more short-wave light than the tungsten lamp on that side of the Mondrian. Area 11 in HDR is *ML* = 55, *Ma* = 62, *Mb* = −15; that is 15 units lighter, 8 units less red and 22 units more blue than ground truth. For this facet the departure from constancy is larger in hue/chroma than in lightness.

The yellow paint samples in Appendix (Data Sheet) 1 show that Areas 68 and 74 appear darker and have less hue/chroma in the LDR scene (distance = 19, 21). In the HDR scene Areas 68 and 74 are both lighter (distance = 14). Area 100 is 24 units darker in HDR, while it appears the same as ground truth in LDR.

The green samples in the LDR scene show that Area 65 is 20 units lighter, and only 5 units lighter in HDR. In LDR areas 50 and 51 are both darker, appear redder and less yellow (distance = 22, 25). In HDR, Area 50 is 20 units lighter, while Area 51 is 40 units darker and 25 units bluer. Area 103 is 18 units darker and 16 units bluer in HDR.

In cyan Area 102 is 20 units darker in HDR. Area 73 is very close to ground truth in both illuminations. Area 53 is about 12 units lighter in both LDR and HDR, and Area 45 is darker by 20 units in LDR; it is 15 units lighter and 16 units bluer in HDR.

In LDR all blue areas were within 10 units of ground truth. In HDR Areas 99 and 47 were darker and bluer (distance = 30, 35).

For the blocks with white paint, the shadows in the LDR caused a drift in lightness of 30 units. In HDR Area 81 showed a distance of 3 units. The same tall thin white facet makes up areas 81, 83, 84, and 85. Area 83 was darker and slightly bluer (distance = 31). Area 84 had light reflected from an adjacent magenta facet. It was darker and more magenta (distance = 42). Area 85 had light reflected from an adjacent yellow facet. It was more yellow (distance = 39).

The magnitude estimate observer data shows that, in general, the color estimates in LDR are closer to ground truth than HDR. Nevertheless, there are areas in the HDR scene that show very small departures from ground truth standard colors. The change in appearance of individual areas depends on the illumination and the other areas in the scene. The sources of illumination, their spatial distributions, and inter-reflections of light from one facet to another, all play a part in generating appearance. One cannot generalize that the surface property (physical reflectance) correlates with the individual facet's sensation. The illumination falling on each individual facet has introduced a considerable variety of changes in sensation. The local spatial properties of illumination (edges and gradients) show significant influence on the hue, lightness and chroma of observed appearances. These measurements provide an extensive dataset for future work in modeling mechanisms that can calculate color sensations, and the variability of color constancy in these 3-D Mondrians. These targets introduce spatial structure in the illumination, and we found greater departures from constancy with increase in illumination structure.

### Artist's watercolor appearances results

**Figure 7:**
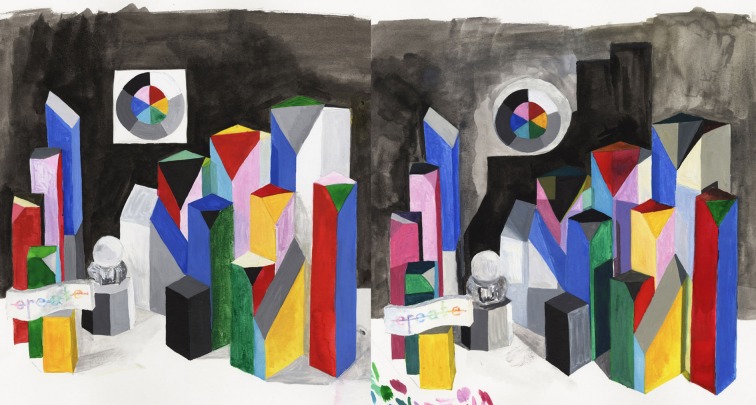
**Photographs of watercolor paintings of LDR/HDR 3-D Mondrians**. The artist matched each painted surface in the surround of all of the other painted surfaces. The matching paint samples in the watercolor can be measured as spectral matches to the scene.

**Figure 8:**
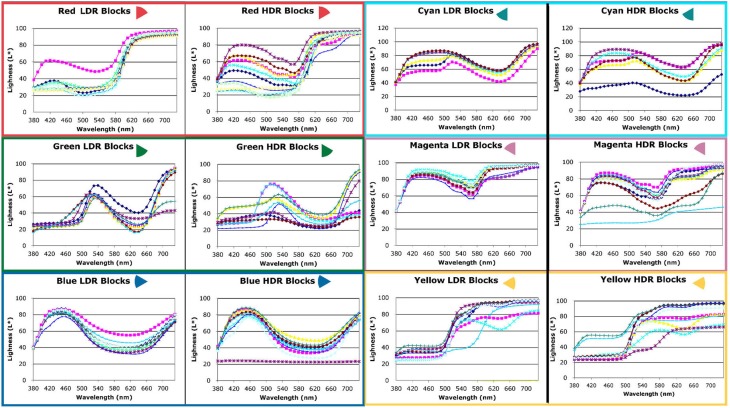
Reflectance spectra scaled by L^*^ of red, green, blue, cyan, magenta, and yellow facets measured from watercolor LDR and HDR paintings.

**Figure 9:**
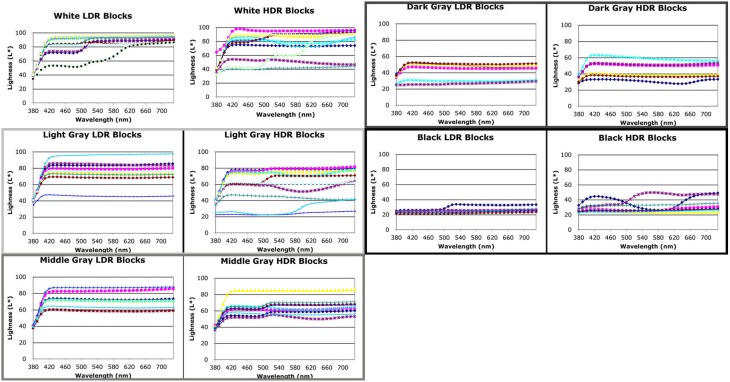
Reflectance spectra scaled by L^*^ of white, grays, and black facets measured from watercolor LDR and HDR paintings.

Figure 7 is a photograph of the watercolor painting of the combined LDR/HDR scene. We made reflectance measurements with a Spectrolino® meter in the center of 104 areas. If the same paint in the scene appeared the same color appearance to the artist, then all the watercolor painting's reflectance plots for these surfaces should superimpose. They do not. The artist selected many different spectra to match the same paint on a number of blocks (Figures 8, 9). Overall, the artist selected a narrower range of watercolor reflectances to reproduce the LDR scene. Many more paint colors are needed to reproduce the HDR scene. Nevertheless, some block facets appeared the same as ground truth, while others showed large departures in their reproduction spectra.

#### Chromatic watercolor reflectances

We plotted the watercolor spectra for all reproductions of the red painted blocks in both LDR and HDR scenes (Figure 8, top row left). In the LDR reproduction, all but one of the facets had very similar measured reflectances. This showed that appearances correlated well with the objects reflectance, with one exception. In the HDR reproduction the painting had a wide variety of measured reflectances, showing that the nonuniform illumination had considerable influence limiting color constancy.

For the green painted blocks we see a wide variety of reproduction spectra in both LDR and HDR paintings. The blue painted blocks had very similar HDR reflectances for all but one of the facets. The LDR reproduction had more variability than the HDR painting. The cyan, and magenta reproductions of the HDR scene showed greater variability in lightness of similar spectra. The yellow reproductions of both showed variability in lightness and spectra.

It is important to study the photographs in Figure 3 and the paintings in Figure 7 to see that these results have more to do with the position of the blocks and their illumination, than with the blocks' paint color. The differences in appearance from ground truth correlate with the spatial structure of the illumination.

#### Achromatic watercolor reflectances

Figure 9 compares the LDR and HDR painting reflectances for the five achromatic value blocks. All departures from a flat spectrum in Figure 9 are examples of hue/chroma introduced by illumination. The white surfaces show considerable variation in lightness and in hue/chroma.

Appendix (Data Sheet) 2 lists the five triplets of radiometric (*X*, *Y*, *Z*) data from these experiments: reflectances of the paints on the blocks; radiances from both the LDR and HDR scenes; and the reflectances of both LDR and HDR watercolor rendering. For the Spectrolino measurements we integrated the reflectance spectra with CIE fundamentals. Then, these values were scaled by L * Equation (4) to approximate the stimulus on the retina. The left triplet of Appendix (Data Sheet) 2 lists the Area Identification Number (Figure 3), the paint, the L * (*X*), L * (*Y*), L * (*Z*) for the paint on the blocks. The right pair of triplets lists the corresponding values for the LDR and HDR watercolor painting. The middle pair of triplets in Appendix (Data Sheet) 2 lists the normalized radiance measurements made with a Konica Minolta CS100 colorimetric telephotometer. We made two measurements (*Y*, *x*, *y*) for each block facet. They were converted to *X*, *Y*, *Z*; averaged and normalized in LDR by the White paint Area 9 measurements (*X* = 284.7, *Y* = 247.5 cd/m^2^, *Z* = 62.8); in HDR by the White paint Area 60 measurements (*X* = 314.2, *Y* = 273 cd/m^2^, *Z* = 88.5). These normalized values were scaled by L * Equation (4).

### Examples of departures from constancy caused by illumination

**Figure 10:**
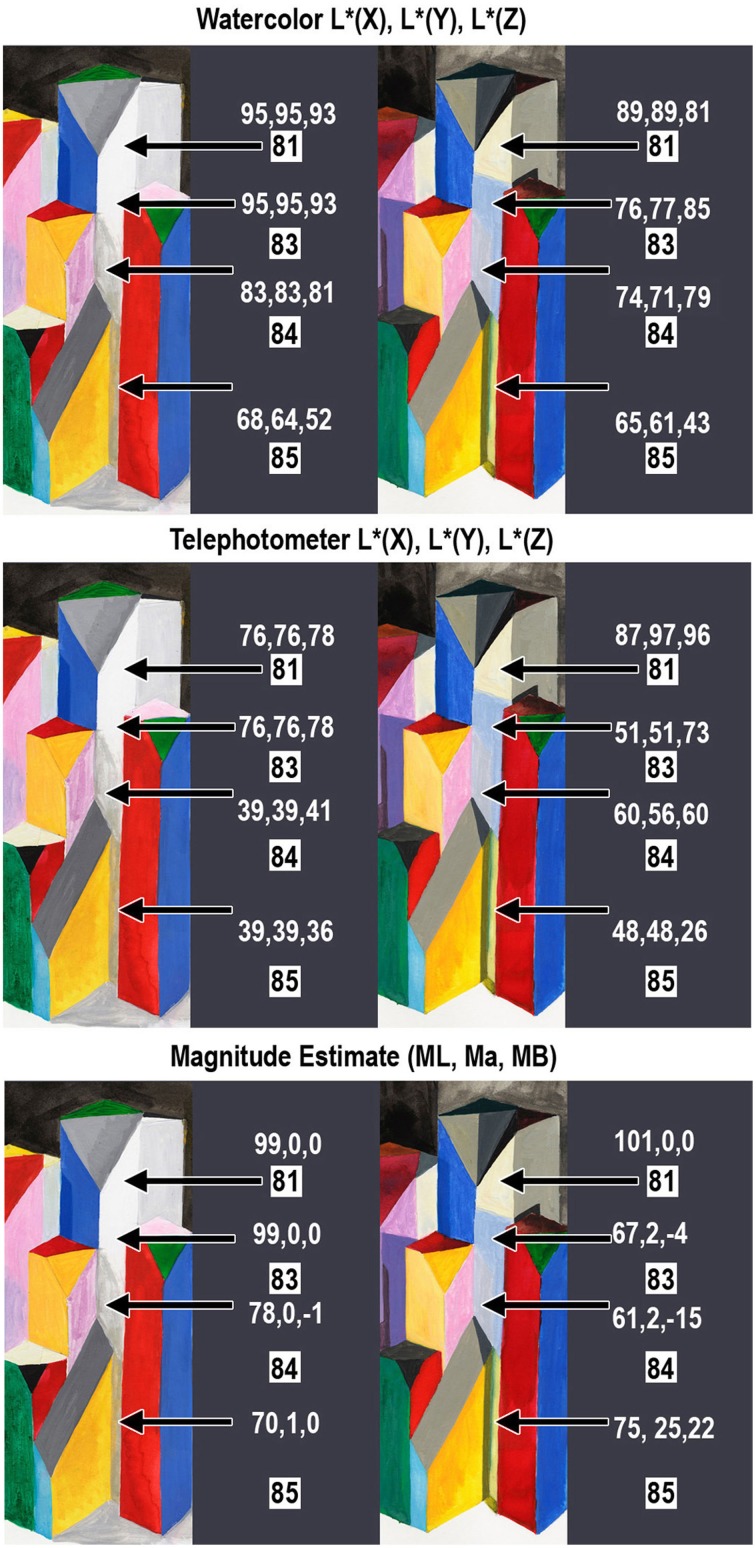
**Measurements of white block in the right of center region of the 3-D Mondrians (left LDR, right HDR)**. All measurements are from a single white block with Areas 81, 83, 84, and 85. The top section shows the watercolor reflectances [L * (*X*), L * (*Y*), L * (*Z*)]. The middle section shows photometer readings from the blocks [L * (*X*), L * (*Y*), L * (*Z*)]; and the bottom section shows average observer magnitude estimates [*ML*, *Ma*, *Mb*].

On the right side of the HDR Mondrian, there is a tall thin white surface. The block's white paint has uniform reflectance values [L * (*X*) = 93, L * (*Y*) = 93, L * (*Z*) = 92)] from the facet's top to bottom [Appendix (Data Sheet) 2]. It illumination is variable because there are different reflections from adjacent blocks. These reflections, from a chromatic block onto an achromatic one, add illumination structure (edges and gradients) to the reflectance structure. The white surface reflectance takes on chromatic appearances, as shown in the spectra in Figure 9 (top row, left) and the photographs in Figure 10.

Figure 10 shows photographic sections of the display, from the LDR and HDR parts. The LDR (left) appearances show light-middle-gray, and dark-middle-gray shadows. The HDR (right) appearances of the single white paint surface show four different colors. The painting shows: white at the top, a blue-gray shadow below it, a pinker reflection and a yellow reflection below that. Shadows and multiple reflections show larger changes in appearance caused by different illumination.

The photographs of the LDR/HDR scene (Figure 3) and the watercolor painting show that the white block in LDR has achromatic shadows. The measurements of watercolor reflectances (Figure 10 top row, left) show that the painter used darker paints to report the darker shadows in LDR. In HDR the painter selected different hues because the white paint on the block was illuminated with a variety of chromatic illuminations.

The measurements of radiances from the white block (Figure 10, middle) show that the shadow spatial structure had achromatic variations in LDR from Appendix (Data Sheet) 2. In HDR, the meter recorded chromatic structure in illumination: a chromatic shift from the two light sources (Area 83) and from multiple reflections (Areas 84, 85). These radiance measurements document the spatial structure in the illumination falling on the uniform reflectance block.

The magnitude estimates of sensations (Figure 10, bottom row) show that the LDR illumination caused changes in lightness, while the HDR illumination caused changes in lightness, chroma and hue Both measurement techniques, watercolor painting and magnitude estimates, show similar results. The changes in illumination falling on this single white block caused relatively sharp edges in light coming to the eye. These retinal images caused observers to report changes in lightness, hue, and chroma. This data supports the observation that the changes in appearance of this white block correlate with the spatial structure in the illumination.

**Figure 11:**
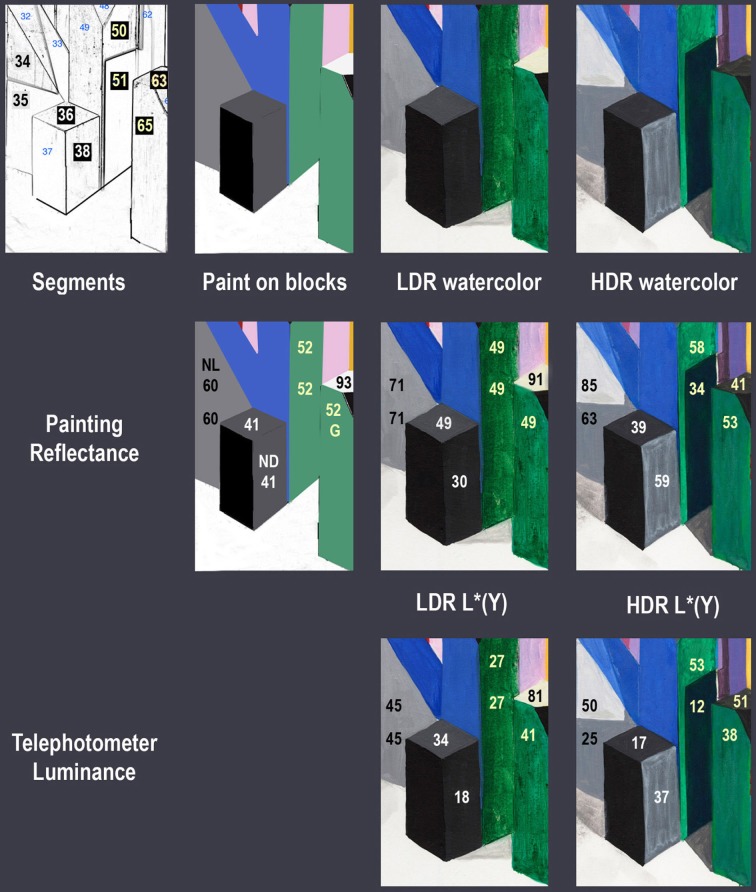
**Measurements of gray, white and green blocks in the center of the 3-D Mondrians**. The top row shows the sketch with Area IDs; the paint used on the blocks; the LDR; and HDR watercolor painting. The middle row shows the Spectrolino® watercolor L * (*Y*) values for these block facets. The bottom row shows the telephotometer L * (*Y*) values for these block facets.

In Figure 11, there is another example of how the illumination structure plays an important role in these color constancy experiments. Figure 11 shows central Mondrian areas surrounding a dark gray and black block (Areas 36 and 38). The captured appearances of the LDR and HDR watercolor renderings have different values from the same paint on that block; Areas 36 and 38 have the same dark gray (G4) paint. They both have reflectance CIE L * (*Y*) values of 41.4. These constant surface reflectances have different appearances in the LDR and HDR portions of the watercolor. In LDR area 36, the top, is lighter [L * (*Y*) = 49] than the side [L * (*Y*) = 30]. In HDR, the top is darker [39], than the side [59].

In the HDR, Area 38 is the lightest of the block's three faces (36, 37, 38), while it is nearly the darkest in the LDR. These changes in appearance correlate with the changes in edges caused by the different illuminations. The bottom row of Figure 11 shows the telephotometer scaled luminances L * (*Y*). LDR area 36 (top) has higher luminance [34], than the side [18]. In HDR, the top has lower luminance [17], than the side [37]. The dark gray facets in Figure 11 illustrate that edges caused by illumination cause substantial change in the appearance of surfaces with identical reflectances.

Facet 63 is another example. It has white paint (L^*^ = 93). In the watercolors it was matched by L^*^ = 91 (LDR) and L^*^ = 41 (HDR).

Facets 50, 51, and 65 all have green paint (L^*^ = 52). The LDR watercolor matches are all equal (L^*^ = 49). The HDR matches are different (L^*^ = 58, 34, 53).

The directions of the changes in appearance are consistent with the directions of changes in illumination on the blocks. Edges in illumination cause substantial changes in appearance. The measurements do not show correlation of appearance with luminance of a local region, rather it demonstrates that change in appearance (sensation) correlates with change in luminance across edges in illumination.

Both magnitude estimates and the artist's rendering give very similar results. Both sets of measurements show that appearance depends on the spatial properties of illumination, as well as reflectance. Edges in illumination cause large changes in appearance, as do edges in reflectance. The magnitude estimates analyze the results in a uniform color space. By definition, distance in this space represents the size of the change in appearance for all hues, lightnesses and hue/chroma. Here we have averaged the estimates of 10 observers for 37 areas in both LDR and HDR. In the second experiment, we analyzed the watercolor painting data for 104 facets for a single observer in a modified colorimetric space. We integrated full spectral data under the color matching functions and scaled them by Equation (4). This color space calculates the retinal spectral response (*X*, *Y*, *Z*) with an approximate correction for intraocular scatter (L^*^) to analyze the retinal response. Both experiments give similar results, but in different color spaces. Further, there are limitations imposed by the gamut of possible colors in the watercolor paints that do not limit the magnitude estimate experiments. The most important comparison is the effect of illumination (LDR vs. HDR) on appearance. Differences in color spaces and small differences caused by experimental techniques are of secondary importance.

**Figure 12:**
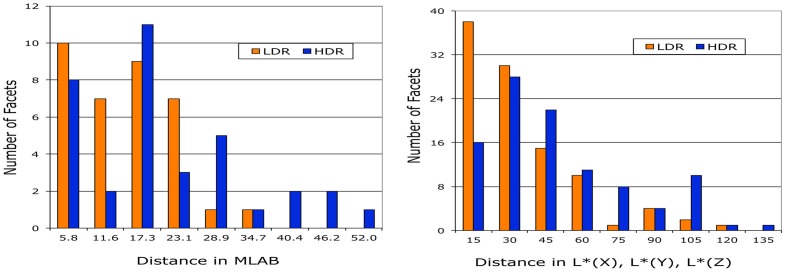
**Comparison of the distributions of LDR and HDR distances from ground truth observed in magnitude estimation and watercolor reflectance experiments**.

Figure 12 plots the distribution of distances between ground truth and observed color for the measurements of appearances (sensations) using the magnitude estimates and the watercolor reflectances. The left graph binned the 37 magnitude estimates of MLAB distances into 9 groups 5.8 units wide. The average LDR magnitude estimate distance from ground truth was 12 ± 8 with a maximum distance of 30 and a minimum of 3. The average HDR magnitude estimate distance from ground truth was 18 ± 13 with a maximum distance of 52 and a minimum of 3. The population LDR distances are greatest close to zero, decreasing with distance. There are no LDR distances near the maximum. The HDR has fewer near zero, with the highest population in the middle of the range. LDR and HDR have different distance distributions.

The right graph (Figure 12) binned the 104 watercolor reflectance distances [in L * (*X*), L * (*Y*), L * (*Z*) space] into 9 groups 15 units wide. The average LDR magnitude estimate distance from ground truth was 27 ± 22 with a maximum distance of 96 and a minimum of 3. The average HDR magnitude estimate distance from ground truth was 42 ± 30 with a maximum distance of 130 and a minimum of 1. Again, the population of LDR distances is greatest close to zero, decreasing with distance. There are no LDR distances in the maximum bin. The HDR has fewer near zero, with the highest population in the middle of the range. The magnitude estimates and watercolor reflectances show similar departures from ground truth.

## Discussion

This paper studies a very simple question. Can illumination change the appearances of paints with the same physical surface reflectance? We are asking this question using a complicated scene with nonuniform illumination falling on 3-D objects; in other words, real scenes, not experimental abstractions. Here we used the placement of objects in the scene to modulate the illumination. We found a complicated answer. There is no universal generalization of our results; such as human vision makes constant surface reflectances appear constant. Rather, we found a wide range of distinct, individual observations. In the LDR, illumination changes appearance some of the time. In the HDR, illumination changes appearance most of the time. Appearance sensations depend on the objects in the scene, their placement, and the spatial structure in the illumination.

Another simple question is whether observer data supports the “discounted illumination” hypothesis. Hering observed that constancy was approximate. The signature of the departures from perfect constancy provides important information about how human vision achieves constancy. The experiments here study how illumination alters the spatial information from the scene. Observer data correlated with spatial structure in the illumination (edges and gradients).

Previous experiments have measured the high correlation between colors in complex scenes with reflectances of the objects' surface (McCann et al., 1976). This good correlation uses Scaled Integrated Reflectance, not the usual spectral surface reflectance curves measured with a narrowband spectral radiometer. This integrated reflectance has L, M, S values that are the product of the surface's spectral reflectance and the L, M, S retinal cone sensitivity functions. [The L reflectance is the ratio of the L cone response to the surface divided by the Lcone response to white paper in the same illumination. The scaling is done by the CIE L^*^ cube root function that approximates a correction for lower reflectances for scatter in the eye (McCann and Rizzi, 2012, ch. 14, 18).] The measured departures from perfect constancy in flat displays in uniform illumination are small, but provide important information about the underlying color constancy mechanism. Further experiments, with many different narrowband spectral illuminants in uniform illumination, showed changes in color appearances are controlled by cone crosstalk, and are inconsistent with cone adaptation theories of constancy, as shown by McCann (2004c, 2005a). These results extended the report by McCann McKee and Taylor that the departures from constancy in uniform illumination were caused by the integrals of reflectance, narrowband illumination and very-broad cone sensitivities. The observed correlation with scaled integrated reflectance was possible because there was no spatial structure in the illumination.

The illumination in the 3-D Mondrian experiments was modulated by the objects in the scene. The departures from perfect constancy measured here in 3-D Mondrians are larger than in uniform illumination. Further, they do not show dependence on the surface reflectances of the paints. We see this in the variability of the distances between appearance and ground truth, and the directions of the color changes in Munsell Space. Each identical colored surface exhibits highly variable changes in appearance. Appearances show dependence on the spatial content of the illumination, as shown in the individual areas described in Figures 10, 11. We also see this in the artist's paint selection used to match appearances in the watercolor. There is great variability in the size and direction of the departures from constancy that correspond to the information about individual areas recorded in the dataset in Appendixes (Data Sheets) 1 and 2.

We found no evidence that visual sensations are the result of illumination detection followed by discounting the illumination. As shown in Figures 10, 11, the changes in appearance of identical reflectances correlate with the departures from uniform illumination. These results, and many other examples documented in this dataset, show that spatial structure in illuminations influences color constancy sensations. Studies comparing the spatial properties of illumination and reflectance show that human form vision processes the spatial content of the illumination the same way it processes the spatial content of the reflectance of objects (McCann, 2000b).

### Color constancy models

As one inspects the color appearances in the LDR and HDR Mondrians one looks for a physical correlate in the scene for color appearances. That correlate is not the XYZ values of a single pixel. The correlate is the spatial relationship of XYZ values with all the other pixels in the rest of the scene. Shadowed regions of the same reflectance paint can have edges created by the illumination. The appearances observed here are consistent with a model that builds colors from image structure.

Spatial comparison algorithms, such as Retinex, use the quanta catch of the cones from the entire field of view as input. Its goal is to calculate the sensations of all areas in the scene. It does this by building the image up from spatial comparisons using the entire image. The model output is equal to the scene's surface reflectance in uniform illumination in flat Mondrians (McCann et al., 1976). It is possible to calculate reflectances using spatial comparisons without ever finding the illumination. Spatial models using 3-D Mondrians will not calculate the paints' reflectances. Instead, we will get a rendition of the scene that treated edges in illumination the same as edges in reflectance. The Retinex spatial model (Frankle and McCann, 1983) shows correlation with reflectance sometimes, (in flat Mondrians), but not all the time (in 3-D Mondrians). A number of computational variations of Retinex spatial processing have been proposed (Frankle and McCann, 1983; Jobson et al., 1997; Marini et al., 1999; McCann, 1999b, 2004a,b, 2005b; Rizzi et al., 2003, 2004; Sobol, 2004; Provenzi et al., 2007, 2008; Bertalmío et al., 2009; Kolås et al., 2011; see McCann and Rizzi, 2012, p. 285–371 for a review).

CIELAB/CIECAM models calculate sensations. They measure the *X*, *Y*, *Z* reflectances of individual pixels and transform them into a new color space. The model uses only two radiance measurements of a single pixel: the radiance coming to the eye, and the illumination falling on that pixel. The ratio of radiances over illumination gives the pixel's reflectance, independent of the content of the rest of the scene. These equations transform the position in color space of the object's reflectance. There is nothing in the calculation that can generate different outputs from identical reflectance inputs. These models predict the same color appearance for all blocks with the same physical reflectance. While useful in analyzing appearances of flat scenes, such as printed test targets, it does not predict appearances with shadows and multiple reflections.

Computer Vision (Computational Color Constancy) has the goal of calculating the object's reflectance, namely the object's intrinsic property. The question here is whether such material recognition models have relevance to human vision. If a computer vision algorithm correctly calculated cone reflectances of flat Mondrians, then one might argue that such processes could happen in human vision (Ebner, 2007). However, the 3-D Mondrians, and other experiments show that illumination affects the observers' responses (Rutherford and Brainard, 2002; Yang and Shevell, 2003). If that same Computer Vision algorithm correctly calculated 3-D Mondrian reflectances, then these calculations would not model their appearances. Computer Vision is a distinct discipline from human vision, with very different objectives. These algorithms are not applicable to human vision.

Surface Perception has the goal of calculating an observer's perceived recognition of a surface's reflectance. In many perception experiments subjects report on the observed properties of objects. The two sides of the lake raft in Figure 1 have different appearances (sensations). Nevertheless, observers recognize that these different appearances are part of the same object in different illumination. Our dataset reports the sensations of constant reflectances in structured illumination. It is not useful in evaluating models that calculate the perception of objects. The observer task was different and the data are not useful in modeling cognition.

### Real paints and lights

In the careful analysis of reflectance and illumination, with its extended dynamic range, there is no room for errors and artifacts introduced by image capture and display technologies. In 1975 we began to study human vision using computer controlled complex image-displays (Frankle and McCann, 1983; McCann and Houston, 1983a). Since then, we have been aware of the need for extensive calibration of electronic imaging devices (McCann and Houston, 1983b). For the experiments in this paper, we chose to fabricate our test scene with real objects painted with exactly the same paints. We chose to use real light sources. We were measured the reflectance of each paint, the *Y*, *x*, *y* of the light coming from the surface, and the full spectra of the paints in the watercolor.

HDR reproduction techniques are widely used today. They include a variety of approaches to render the appearance of HDR scenes in LDR Media (Frankle and McCann, 1983; McCann, 1988, 2004a,b, 2007). Multiple exposure techniques are used to improve photographic reproductions (Debevec and Malik, 1997; Reinhard et al., 2006). Nevertheless, multiple exposures do not record accurate scene radiances. Rather they record the sum of the scene radiance and the undesired veiling glare from the camera and its optics. Glare is image dependent, and cannot be corrected by calibration (McCann and Rizzi, 2007; Rizzi and McCann, 2009). Scene information and glare cannot be separated without independent radiance measurements of the scene.

Similarly, there are great difficulties in error-free rendering the information stored in computer memory on a print, or display device. Extensive calibrations of all image areas throughout the full 3-D color space are needed to avoid hardware limitations. The hardware systems that convert digits to light have many operations that alter the light coming to the eye from the expected value to a different device-dependent value. The digital image stored in computer memory is continuously sent, via a graphics card, to the display pixel (refreshed at the rate specified by the hardware). The physical characteristics of the display (spectral emission, number and size of pixels); the time budget (refresh rate and response times), the image processing in the graphics card; and the circuitry in the display all influence the display's light output at each pixel. The amount of light output does not always correspond to image digits in computer memory. A good example is that the EMF of the display signals in the screen wiring introduces image-dependent color shifts (Feng and Daly, 2005). Hardware systems introduce image-dependent transformations of the input signals that on average improve the display's appearance (Feng and Yoshida, 2008). HDR displays with two active light modulators introduce even more complexity with high-resolution LCDs, and low-resolution LEDs. The system integrates the two images with complex, proprietary, spatial filtering of the image data (Seetzen et al., 2004). It is not a simple matter to verify the accuracy of a display over its entire light-emitting surface, for all light levels, for its entire 3-D 24-bit color space. The combination of reflectances (range = 100:1), and illuminations (range = 100:1) require great precision over a range of 10,000:1. Rather than calculate the combined effects of reflectances and illumination for an image-dependent display device, and verify its accuracy with calibration measurements, we chose to use real lights and paints for this analysis.

### Dataset applications

We made RAW format digital photographs of the LDR and HDR parts of the display using a Leaf Aptus digital sensor in a Mamiya body camera. We used multiple exposures to verify the camera's range of linear response. Using the KM spotmeter readings we calibrated the linear portion of RAW camera digits to convert to XYZ data. The next steps convert XYZ to cone response and then use the human glare spread function (Vos and van den Berg, 1999) to calculate the cone quanta catch of the retinal image that includes the veiling glare of intraocular scatter. Calibrated digital images of the arrays of scene radiance and cone quanta catches will be added to the dataset reported here. These images can be used as the calibrated input to models of color appearance and object intrinsic properties. The details of image calibration and model analysis are beyond the scope of this paper.

### Conclusions

We measured the sensation appearances of two identical arrays of 3-D objects in nearly uniform (LDR) and nonuniform (HDR) illumination. They were viewed in the same room at the same time. All flat facets were painted with one of 11 paints. We used two different techniques to measure the appearances of these constant reflectance paints. In the first, observers made magnitude estimates of changes in Munsell notation; in the second we measured the reflectance spectra of an artist's watercolor rendition of both scenes. Departures from perfect color constancy are the signature of the underlying mechanism. Both magnitude estimates and watercolor reflectances showed that departures depended on the spatial structure measured in the illumination. The dataset reported here provides measurements of radiances and sensations in complex scenes for future analysis by computational models of appearance. If a computer algorithm discounted the illumination, and succeeded in accurately calculating an object's reflectance, then that algorithm would not predict observed sensations in real-life scenes with complex nonuniform illumination.

### Conflict of interest statement

The authors declare that the research was conducted in the absence of any commercial or financial relationships that could be construed as a potential conflict of interest.
