# Supplementary material for: Reflectance, illumination, and appearance in color constancy
Source: Front Psychol. 2014 Jan 24;5:5. doi: 10.3389/fpsyg.2014.00005 (PMC3901009; doi:10.3389/fpsyg.2014.00005)
Supplement: Supplementary file 1 [file DataSheet1.PDF]

Appendix 1

Low Dynamic Range

Paint	area#	ML mean	Ma mean	Mb mean	Δ ML	Δ Ma	Δ Mb	MLAB distance	Angle (deg) (Ma vs. ML)	Angle (Ma vs. Mb)
Paint	area#	40	70	7						
R1	97	44±2	67±6	5.8±1	4.5	-3.1	-1.5	5.7	125	206
R2	56	52±4	64±4	5.5±2	12.0	-5.4	-1.8	13.3	114	199
R3	55	52±4	64±4	5.5±2	12.0	-5.4	-1.8	13.3	114	199
R4	92	49±2	62±2	4.8±3	8.8	-7.3	-2.5	11.7	130	199
R5	11	43±2	64±5	3.2±4	3.0	-5.4	-4.1	7.4	151	217
				range	9	4	3	7.6		
	paint	80	15	68						
Y1	100	80±2	16±1	69±2	0.5	1.1	-3.5	3.7	24	287
Y2	68	69±4	12±2	56±4	-11.4	-3.1	-15.4	19.4	255	259
Y3	69	85±4	15±1	68±1	5.4	0.7	-9.1	10.6	83	274
Y4	19	78±3	14±0.4	66±2	-2.3	-0.7	-6.3	6.7	253	264
Y5	74	64±4	13±1	59±2	-16.5	-2.0	-13.5	21.4	263	262
				range	22	4	12	17.7		
		50	-40	45						
G1	103	57±4	- 36±3	45±1	6.8	3.6	0.6	7.7	62	9
G2	50	31±5	- 30±4	38±4	-18.7	10.5	-6.3	22.4	299	329
G3	51	33±6	- 29±4	38±4	-21.8	10.7	-6.3	25.1	296	330
G4	65	70±5	- 35±4	45±1	19.5	4.6	0.6	20.0	77	7
G5	16	52±3	- 37±4	45±1	1.6	2.9	0.8	3.4	29	15
				range	41	8	7	21.7		
Paint	area#	60	-37	-16						
C1	102	62±1	- 38±1	- 14±2	1.8	-0.9	2.0	2.8	117	115
C2	53	72±2	- 37±1	- 14±2	12.3	-0.3	2.2	12.5	91	97
C3	45	40±5	- 35±2	- 10±3	-20.3	1.8	6.6	21.4	275	75
C4	73	62±1	- 37±1	- 13±2	1.8	-0.6	3.2	3.7	107	100
				range	-22	0	5	18.5		
Paint	area#	40	-3	-59						
B1	99	43±2	- 3±0	- 59±1	2.5	0.0	4.5	5.1	90	90
B2	47	48±3	- 3±0	- 59±2	7.6	0.0	4.8	9.0	90	90
B3	49	50±3	- 3±0.2	- 57±3	10.2	0.1	7.3	12.5	89	89
B4	33	53±4	- 3±0	- 59±1	9.0	0.0	4.8	10.2	90	90
				range	8	1	3	7.4		
Paint	area#	60	34	-37						
M1	96	63±1	34±0.5	-37±1	2.6	-2.2	-1.2	3.6	130	208
M2	59	69±2	34±0.7	-38±1	10.0	-5.8	-3.2	12.0	120	209
M3	90	73±2	33±2	-37±2	14.5	-4.6	-1.8	15.3	108	201
M4	44	71±3	36±1	-38±1	12.6	-2.9	-4.0	13.6	103	234
M5	70	52±3	32±1	-37±2	-12.3	-7.3	-0.7	14.3	239	185
M6	6	43±7	32±2	-35±2	-16.9	-10.5	-0.1	19.9	238	181
				range	27	8	4	16.3		
		100.0	0.0	0.0						
W-1	81	88±7	6±6	0±0	-1.5	0.0	0.0	1.5	270	
W-2	83	88±7	6±6	0±0	-1.5	0.0	0.0	1.5	270	
W-3	84	69±8	2±2	0±0	-22.2	0.1	-0.5	22.2	270	277
W-4	85	65±6	8±8	7±7	-29.7	0.5	0.4	29.7	271	39
				range	29	1	0	28.2		
		75.0	0.0	0.0						
G1-1	101	75±0	0	0	0	0	0	0		
		40.0	0.0	0.0						
G3-1	98	41±2	0.0	0.0	1.3	0.0	0.0	1.3	90	
G3-2	36	45±2	0.5±0.3	- 0.2±0.2	5.0	0.5	-0.2	3.7	84	339
G3-3	38	27±5	0.7±0.7	- 0.4±0.5	-13.6	0.7	-0.4	8.2	273	331
				range	19	1	0	6.9		

High Dynamic Range

Paint	area#	ML mean	Ma mean	Mb mean	Δ ML	Δ Ma	Δ Mb	MLAB distance	Angle (Ma vs. ML)	Angle (Ma vs. Mb)
Paint	area#	40	70	7						
R1	97	27±4	59±4	4±2	-12.9	-10.2	*-3.0*	16.7	232	196
R2	56	26±4	50±8	1±2	-13.5	-19.6	*-6.3*	24.6	214	198
R3	55	52±3	76±4	9±1	11.8	6.0	*1.7*	13.3	63	16
R4	92	44±2	70±2	7±1	4.1	0.7	*-0.5*	4.2	80	325
R5	11	55±3	62±6	- 15±5	15.0	-7.8	*-22.2*	27.9	118	251
				range	25	26	24	28.8		
Paint	area#	80	15	68						
Y1	100	56±6	15±1	61±4	-24.1	0.03	-7.8	25.3	270	270
Y2	68	94±4	14±1	68±4	14.4	-0.1	-0.6	14.4	91	257
Y3	69	85±3	14±1	67±6	5.4	-0.4	-1.6	5.6	94	257
Y4	19	89±5	18±3	70±7	8.8	3.6	1.4	9.6	68	22
Y5	74	94±5	14±2	67±4	14.4	-0.3	-1.2	14.5	91	257
				range	38	2	7	15.7		
	area#	50	-40	45						
G1	103	32±6	- 27±4	28±4	-17.6	12.7	-16.2	27.1	306	308
G2	50	70±5	- 41±3	46±4	20.0	-1.3	1.7	20.1	94	127
G3	51	10±3	- 18±6	20±7	-40.0	22.1	-24.8	52.0	299	312
G4	65	55±1	- 41±1	45±1	4.5	-0.3	0.3	4.5	94	135
G5	16	94±5	- 39±4	40±3	12.2	1.5	-4.4	13.0	83	289
				range	60	23	-26	47.5		
Paint	area#	60	-37	-16						
C1	102	40±5	-27±3	-9±2	-20.2	-9.4	6.9	23.3	245	144
C2	53	70±4	-37±1	- 14±3	12.1	1.2	1.9	12.3	84	58
C3	45	75±3	-39±2	0±4	14.6	2.9	16.3	22.0	79	80
C4	73	62±1	-36±0	- 14±2	1.5	-0.1	2.4	2.8	94	93
				range	1	0	2	20.6		
			7.2	-48.9						
Paint	area#	40	-3	-59						
B1	99	18±3	-1±1	- 39±7	-21.7	-2.8	20.0	29.7	263	98
B2	47	14±4	-2±1	- 35±8	-25.6	-4.6	23.6	35.1	260	101
B3	49	47±4	-4±0	- 62±5	6.5	2.6	-3.1	7.6	68	309
B4	33	36±9	-2±1	- 58±4	-4.1	-3.1	0.5	5.2	233	171
				range	32	2	23	29.9		
Paint	area#	60	34	-37						
M1	96	39±4	29±2	- 33±2	-18.0	0.0	*4.4*	18.5	270	91
M2	59	61±3	34±2	- 41±3	0.0	0.0	*-3.5*	3.5	180	269
M3	44	54±7	19±2	- 36±3	-8.0	-15.3	*1.6*	17.3	208	174
M4	6	63±4	37±3	- 34±3	3.0	3.2	*3.4*	5.6	43	47
M5	90	29±7	15±3	- 19±4	-40.0	-21.4	*3.5*	45.5	242	171
M6	70	72±4	33±1	- 36±3	12.0	0.0	*1.4*	12.1	90	92
				range	52	25	7	42.0		
	area#	100.0	0.0	0.0						
W-1	81	103±5	0.0	0.0	3.3	0.0	0.0	3.3	90	
W-2	83	70±6	2±1	- 4±3	-30.3	2.2	-3.6	30.6	274	301
W-3	84	61±6	3±1	- 13±3	-39.2	2.7	-13.4	41.5	274	281
W-4	85	75±4	23±5	18±4	-25.0	23.0	18.0	38.5	313	38
				range	42	23	26	54.5		
		75.0	0.0	0.0						
G1-1	101	58±2	2±2	1±1	-16.5	1.8	0.6	16.6	276	19
		40.0	0.0	0.0						
G3-1	98	21±3	0.0	0.0	-19	0.0	0.0	19.4	270	
G3-2	64	27±6	0.0	0.0	-13	0.0	0.0	13.0	270	
G3-3	38	54±3	0.0	0.0	13	0.0	0.0	13.2	90	
				range	33	0.0	0.0	6.4		
