# Supplementary material for: Reflectance, illumination, and appearance in color constancy
Source: Front Psychol. 2014 Jan 24;5:5. doi: 10.3389/fpsyg.2014.00005 (PMC3901009; doi:10.3389/fpsyg.2014.00005)
Supplement: Supplementary file 2 [file DataSheet2.PDF]

Appendix 2

		Scene Radiance								
		Paint Sample			3-D Mondrians LDR KM100			3-D Mondrians HDR KM100		
Paint	Area	L*(X)	L*(Y)	L*(Z)	L*(X)	L*(Y)	L*(Z)	L*(X)	L*(Y)	L*(Z)
R	7	60.5	49.1	35.9	46.7	36.6	23.4	41.6	32.8	28.1
R	10	60.5	49.1	35.9	90.6	77.2	59.7	48.1	40.8	50.9
R	11	60.5	49.1	35.9	57.5	46.4	30.7	56.8	48.5	65.1
R	13	60.5	49.1	35.9	57.5	46.4	30.7	34.2	28.8	46.0
R	30	60.5	49.1	35.9	58.2	46.4	29.5	55.8	46.7	59.7
R	31	60.5	49.1	35.9	58.2	46.4	29.5	40.8	35.4	54.7
R	55	60.5	49.1	35.9	58.4	47.1	31.4	65.9	52.3	35.3
R	56	60.5	49.1	35.9	58.4	47.1	31.4	23.8	19.3	26.0
R	66	60.5	49.1	35.9	38.2	29.9	18.7	82.9	66.5	39.5
R	67	60.5	49.1	35.9	51.2	41.0	28.0	45.9	40.3	37.0
R	92	60.5	49.1	35.9	52.8	44.1	16.3	67.6	53.6	36.7
R	97	60.5	49.1	35.9	58.4	46.9	30.2	9.3	5.9	3.6
Y	19	81.5	77.4	26.6	55.0	52.6	24.2	58.3	56.3	22.3
Y	20	81.5	77.4	26.6	56.4	53.8	26.5	51.9	50.0	23.3
Y	43	81.5	77.4	26.6	69.9	67.0	27.2	29.9	32.5	45.0
Y	54	81.5	77.4	26.6	75.7	72.5	29.6	19.5	18.3	14.0
Y	68	81.5	77.4	26.6	55.3	53.0	20.7	98.1	94.2	33.2
Y	69	81.5	77.4	26.6	74.5	71.6	29.6	81.2	79.7	46.9
Y	74	81.5	77.4	26.6	43.9	40.9	15.2	103.3	99.0	34.0
Y	75	81.5	77.4	26.6	31.1	29.8	14.3	46.4	44.2	12.3
Y	100	81.5	77.4	26.6	73.0	69.2	28.6	13.2	12.1	2.7
G	14	42.1	51.7	33.4	46.5	54.0	40.8	31.7	30.9	41.4
G	16	42.1	51.7	33.4	25.3	31.6	23.9	29.8	40.8	39.2
G	17	42.1	51.7	33.4	25.3	31.6	23.9	13.9	18.8	20.7
G	50	42.1	51.7	33.4	24.6	27.3	23.0	41.8	52.7	40.8
G	51	42.1	51.7	33.4	24.6	27.3	23.0	10.3	12.1	18.5
G	65	42.1	51.7	33.4	34.0	41.1	31.9	29.2	37.8	28.5
G	76	42.1	51.7	33.4	35.6	43.2	34.5	14.8	16.0	16.4
G	91	42.1	51.7	33.4	25.5	34.3	32.1	43.8	55.8	37.5
G	103	42.1	51.7	33.4	30.8	38.8	28.3	2.4	4.4	3.8
C	39	54.2	62.2	67.7	45.9	52.7	66.4	14.0	16.0	31.5
C	45	54.2	62.2	67.7	23.4	27.5	41.4	52.9	60.7	81.5
C	52	54.2	62.2	67.7	30.4	36.1	50.2	44.7	53.4	79.1
C	53	54.2	62.2	67.7	46.6	53.4	67.7	51.0	58.9	102.5
C	73	54.2	62.2	67.7	40.8	46.4	57.7	38.0	44.4	57.4
C	102	54.2	62.2	67.7	38.3	44.3	58.2	3.9	5.6	11.2
B	2	43.7	44.7	66.7	31.0	32.4	54.9	35.5	35.7	87.9
B	3	43.7	44.7	66.7	26.4	27.5	47.5	22.7	23.7	45.0
B	32	43.7	44.7	66.7	32.4	33.6	56.4	37.2	37.6	87.4
B	33	43.7	44.7	66.7	32.4	33.6	56.4	22.2	21.0	69.1
B	47	43.7	44.7	66.7	36.2	38.5	65.7	22.5	25.6	55.9
B	49	43.7	44.7	66.7	35.8	37.6	63.6	38.8	38.9	95.5
B	80	43.7	44.7	66.7	34.9	36.0	57.9	39.9	41.2	77.4
B	82	43.7	44.7	66.7	20.9	21.2	37.6	40.9	42.3	67.3
B	93	43.7	44.7	66.7	34.9	36.3	59.7	48.2	50.8	82.6
B	99	43.7	44.7	66.7	32.1	33.8	58.3	1.4	1.8	10.7
M	5	67.8	61.7	70.3	66.4	59.7	64.7	51.2	44.8	50.2
M	6	67.8	61.7	70.3	69.9	62.5	68.5	67.7	62.4	110.0
M	44	67.8	61.7	70.3	68.9	61.4	66.9	67.5	62.0	107.0
M	46	67.8	61.7	70.3	68.9	61.4	66.9	48.6	45.4	96.8
M	59	67.8	61.7	70.3	69.3	61.8	66.8	70.3	64.1	96.3
M	61	67.8	61.7	70.3	69.3	61.8	66.8	45.4	43.4	84.5
M	62	67.8	61.7	70.3	36.4	31.6	33.1	62.6	55.6	59.9
M	70	67.8	61.7	70.3	36.6	32.3	36.4	93.9	83.6	88.0
M	90	67.8	61.7	70.3	76.1	68.9	74.6	34.6	32.7	32.4
M	96	67.8	61.7	70.3	62.9	56.5	62.6	10.7	8.7	12.9
W	9	93.2	93.4	92.0	100.0	100.0	100.0	35.5	34.0	53.2
W	24	93.2	93.4	92.0	49.1	49.1	50.2	68.0	68.2	69.5
W	28	93.2	93.4	92.0	49.0	48.8	50.1	57.2	57.2	64.1
W	29	93.2	93.4	92.0	49.0	48.8	50.1	25.2	24.9	29.0
W	57	93.2	93.4	92.0	73.8	73.8	75.6	31.2	31.4	38.5
W	60	93.2	93.4	92.0	39.6	38.3	42.7	100.0	100.0	100.0
W	63	93.2	93.4	92.0	81.4	80.7	78.2	51.3	50.5	45.4
W	81	93.2	93.4	92.0	76.4	76.4	77.5	86.8	87.4	96.3
W	83	93.2	93.4	92.0	76.4	76.4	77.5	51.1	51.5	73.6
W	84	93.2	93.4	92.0	39.6	38.7	40.7	63.0	56.5	60.7
W	85	93.2	93.4	92.0	39.0	38.6	35.6	48.1	46.8	26.1
W	95	93.2	93.4	92.0	80.8	81.0	83.1	16.6	16.6	20.5
NL	1	72.5	73.1	74.6	58.8	59.3	62.6	32.4	32.7	35.3
NL	15	72.5	73.1	74.6	55.6	55.8	58.2	48.0	48.4	51.9
NL	23	72.5	73.1	74.6	39.1	39.2	41.3	61.2	61.5	62.1
NL	26	72.5	73.1	74.6	54.4	54.7	58.2	47.9	49.8	81.9
NL	27	72.5	73.1	74.6	54.4	54.7	58.2	40.1	42.6	81.0
NL	77	72.5	73.1	74.6	45.4	45.7	47.9	69.0	69.7	69.9
NL	78	72.5	73.1	74.6	45.4	45.7	47.9	13.6	13.9	21.9
NL	86	72.5	73.1	74.6	24.7	25.2	26.5	13.0	12.9	13.5
NL	87	72.5	73.1	74.6	63.1	63.7	66.1	25.8	26.5	25.9
NL	101	72.5	73.1	74.6	60.1	60.6	64.0	9.2	9.3	12.8
NM	18	59.5	60.1	61.1	47.8	48.2	49.8	30.8	31.3	45.5
NM	25	59.5	60.1	61.1	36.0	36.2	38.4	26.3	26.4	32.7
NM	34	59.5	60.1	61.1	44.9	45.1	47.0	48.7	50.3	73.6
NM	35	59.5	60.1	61.1	44.9	45.1	47.0	24.8	25.1	30.0
NM	71	59.5	60.1	61.1	28.5	28.1	29.7	26.4	25.7	29.2
NM	72	59.5	60.1	61.1	28.5	28.1	29.7	70.9	71.1	69.9
NM	88	59.5	60.1	61.1	62.5	63.0	65.7	69.1	69.7	68.4
NM	89	59.5	60.1	61.1	62.5	63.0	65.7	20.9	19.7	20.0
NM	104	59.5	60.1	61.1	48.4	48.8	51.3	5.7	5.7	8.3
ND	4	41.0	41.4	43.3	44.1	44.8	50.4	22.8	21.1	45.4
ND	22	41.0	41.4	43.3	18.8	19.1	21.5	23.5	23.9	26.3
ND	36	41.0	41.4	43.3	33.8	33.9	39.3	16.6	16.6	39.2
ND	38	41.0	41.4	43.3	19.4	18.2	18.8	36.2	36.7	38.9
ND	48	41.0	41.4	43.3	28.0	28.4	31.5	39.7	40.4	48.1
ND	98	41.0	41.4	43.3	34.6	35.0	38.2	1.9	2.1	4.5
K	8	26.0	26.0	26.5	35.4	34.6	36.3	28.3	25.9	47.0
K	12	26.0	26.0	26.5	16.8	15.9	18.0	19.7	17.8	22.7
K	21	26.0	26.0	26.5	12.2	11.6	12.1	13.5	10.9	8.9
K	37	26.0	26.0	26.5	19.6	19.6	20.5	12.8	13.0	19.4
K	40	26.0	26.0	26.5	22.8	22.6	22.5	17.9	18.2	23.7
K	41	26.0	26.0	26.5	22.8	22.6	22.5	8.8	9.4	18.2
K	64	26.0	26.0	26.5	14.7	14.6	15.3	26.3	26.0	25.5
K	79	26.0	26.0	26.5	23.2	23.3	24.9	10.6	10.7	15.0
K	94	26.0	26.0	26.5	19.0	19.0	20.7	0.3	0.1	3.2

		Painting					
		LDR Watercolor Spectralino			HDRWatercolor Spectralino		
Paint	Area	L*(X)	L*(Y)	L*(Z)	L*(X)	L*(Y)	L*(Z)
R	7	61.0	47.5	33.3	56.0	45.3	46.3
R	10	74.0	63.9	58.8	61.6	53.9	59.8
R	11	62.8	50.7	33.8	69.9	58.9	63.6
R	13	62.8	50.7	33.8	57.7	48.2	52.2
R	30	57.2	45.7	27.4	78.6	71.5	78.3
R	31	57.2	45.7	27.4	66.4	59.6	65.8
R	55	60.7	48.7	27.7	57.3	44.3	23.8
R	56	60.7	48.7	27.7	47.4	38.1	33.2
R	66	58.0	44.7	25.9	61.9	48.3	23.5
R	67	57.9	44.0	28.5	53.5	41.6	22.8
R	92	62.0	49.4	27.2	56.8	42.7	28.7
R	97	55.9	44.7	26.0	51.0	41.1	27.7
Y	19	82.7	76.1	28.7	85.7	81.6	29.6
Y	20	68.8	65.5	28.6	71.6	69.3	25.4
Y	43	63.8	64.1	26.7	55.7	53.8	30.1
Y	54	86.2	83.6	38.9	50.3	44.1	23.9
Y	68	85.1	80.8	35.4	85.3	81.6	30.3
Y	69	85.2	81.4	42.7	87.1	84.6	55.5
Y	74	81.8	78.0	34.2	82.1	76.7	28.1
Y	75	63.7	53.3	36.3	71.4	71.9	51.6
Y	100	82.0	77.0	26.7	78.5	73.4	28.3
G	14	51.6	58.4	29.4	28.2	31.1	36.2
G	16	34.6	42.7	25.0	46.3	59.2	37.8
G	17	34.6	42.7	25.0	43.9	49.1	49.4
G	50	40.4	49.0	30.3	44.3	57.8	38.2
G	51	40.4	49.0	30.3	30.0	33.7	35.9
G	65	37.5	48.6	33.8	47.9	53.2	49.5
G	76	38.3	44.8	24.9	27.8	29.7	31.9
G	91	38.2	46.7	26.7	30.4	37.2	23.6
G	103	36.3	47.6	33.4	35.3	42.2	30.4
C	39	66.4	71.9	65.7	28.5	32.4	35.6
C	45	52.8	58.7	57.0	70.8	75.4	71.9
C	52	63.3	68.8	71.8	55.3	61.0	65.2
C	53	67.3	72.2	82.1	63.0	67.8	80.6
C	73	68.7	73.4	81.7	74.0	78.1	87.0
C	102	69.9	75.0	83.9	57.1	63.6	70.6
B	2	54.6	53.9	84.1	54.1	53.3	84.2
B	3	63.6	64.4	84.5	49.9	47.8	82.7
B	32	50.7	49.3	80.8	59.4	60.0	83.4
B	33	50.7	49.3	80.8	49.1	49.6	74.2
B	47	48.5	46.8	79.3	22.8	22.8	23.9
B	49	52.7	52.5	78.9	53.4	53.5	80.1
B	80	52.4	52.4	78.3	56.7	56.9	85.6
B	82	45.5	45.2	73.1	47.8	48.3	75.3
B	93	55.9	56.3	79.7	50.8	50.4	78.4
B	99	54.5	54.1	83.5	41.4	41.0	69.8
M	5	85.7	81.6	87.6	72.6	66.1	73.2
M	6	76.0	73.0	83.2	83.4	78.9	85.4
M	44	86.4	82.6	87.9	73.8	70.8	80.3
M	46	89.3	86.5	90.4	75.5	72.1	83.1
M	59	81.7	76.8	84.4	74.6	70.4	81.9
M	61	81.7	76.8	84.4	55.9	53.6	73.1
M	62	79.1	77.4	86.4	43.3	41.7	46.8
M	70	75.3	70.2	80.5	79.9	74.6	81.7
M	90	87.4	83.3	87.6	34.5	31.6	26.9
M	96	87.2	83.7	88.6	75.1	68.8	78.0
W	9	85.7	85.7	71.6	73.3	73.4	74.4
W	24	92.3	92.4	91.5	95.1	94.9	95.9
W	28	93.0	93.0	90.9	87.2	87.6	87.9
W	29	93.0	93.0	90.9	78.4	79.0	81.9
W	57	85.9	85.6	73.3	50.9	51.7	53.3
W	60	84.1	84.8	83.7	88.4	88.7	78.8
W	63	91.1	91.1	83.8	41.4	41.2	39.8
W	81	94.7	94.8	93.4	89.7	89.9	81.3
W	83	94.7	94.8	93.4	75.5	76.5	85.3
W	84	83.0	83.1	81.7	74.1	71.3	78.6
W	85	69.4	64.3	52.4	65.5	61.3	42.5
W	95	95.8	95.9	94.2	89.6	89.8	89.8
NL	1	83.4	83.3	83.0	78.3	78.5	77.3
NL	15	78.7	78.7	78.9	78.7	78.8	76.1
NL	23	73.7	73.9	74.8	74.9	74.9	73.3
NL	26	84.3	84.5	85.3	71.7	72.3	74.8
NL	27	84.3	84.5	85.3	53.8	54.6	59.6
NL	77	71.8	71.8	72.5	68.7	68.8	60.7
NL	78	68.1	68.1	68.8	43.6	44.1	46.1
NL	86	45.3	45.3	46.5	23.8	23.1	22.4
NL	87	96.1	96.2	94.4	29.7	26.6	24.4
NL	101	75.5	75.6	76.3	60.7	60.7	61.9
NM	18	72.3	72.4	73.4	57.8	57.9	53.7
NM	25	82.9	82.8	82.0	61.1	61.0	61.6
NM	34	70.9	70.9	71.7	84.5	84.6	84.3
NM	35	70.9	70.9	71.7	62.2	62.5	65.2
NM	71	58.6	58.6	59.6	51.6	52.7	51.7
NM	72	58.6	58.6	59.6	66.7	66.8	62.4
NM	88	86.8	86.8	86.5	69.2	69.4	64.8
NM	89	86.8	86.8	86.5	65.6	65.7	61.7
NM	104	62.7	62.7	63.9	55.3	55.3	56.9
ND	4	48.2	48.6	51.1	29.7	30.3	32.9
ND	22	45.1	45.1	46.3	50.3	50.2	51.6
ND	36	49.0	49.0	50.3	38.7	38.6	39.9
ND	38	29.6	29.5	30.2	58.6	58.9	62.1
ND	48	26.7	26.5	25.4	51.3	51.2	52.5
ND	98	50.6	50.6	51.8	36.4	36.3	37.6
K	8	31.8	32.2	26.1	32.2	30.5	42.5
K	12	25.1	25.0	24.3	26.5	25.8	26.4
K	21	23.2	22.9	22.4	23.3	23.3	24.4
K	37	23.3	23.1	22.6	25.6	25.4	25.5
K	40	6.2	6.3	12.2	46.1	46.3	33.1
K	41	21.9	21.7	21.2	26.0	25.7	25.4
K	64	25.0	24.7	24.2	32.9	32.8	32.3
K	79	2.1	2.2	3.9	25.4	25.2	24.9
K	94	23.7	23.4	22.9	24.2	23.9	23.3
